# Teacher identity as a mediator in the relationship between self-efficacy and possible selves

**DOI:** 10.3389/fpsyg.2026.1787692

**Published:** 2026-09-01

**Authors:** Ceyhun Kavrayıcı

**Affiliations:** Department of Educational Sciences, Faculty of Education, Anadolu University, Eskişehir, Türkiye

**Keywords:** expected possible selves, feared possible selves, pre-service teachers, self-efficacy, teacher identity

## Abstract

The study investigates the role of teacher identity as a mediator in the relationship between self-efficacy and possible selves. We employed a cross-sectional study design in this research. Participants consisted of 511 volunteer pre-service teachers (70.5% female) attending a university in the Central Anatolia region of Türkiye. After providing written informed consent, participants responded to the Teacher’s Sense of Efficacy Scale, the Teacher Identity Scale, and the New Teacher Possible Selves Questionnaire. To test the hypothesized indirect effects, data were analyzed using Pearson correlation and mediation analyses via the SPSS PROCESS macro (Model 4) with 5,000 bootstrap samples. The analysis indicated a positive correlation between self-efficacy and expected possible selves, while a negative correlation was observed with feared possible selves. Besides, teacher identity was positively correlated with expected possible selves and negatively correlated with feared possible selves. Mediation analysis indicated that the findings are consistent with a mediation model, supporting an indirect association pattern between self-efficacy and expected possible selves through teacher identity. Furthermore, the analysis revealed a similar indirect association pattern for the relationship between self-efficacy and feared possible selves. These findings offer valuable insights for developing strategies to strengthen expected possible selves in pre-service teachers. They also point to potential pathways for mitigating the intensity of feared possible selves.

## Introduction

A primary objective of teacher education programs is to foster the psychological and professional growth of pre-service teachers, ensuring they are equipped to navigate the complexities of the classroom. Among the most critical constructs cultivated during this formative period are self-efficacy, professional identity, and the envisioning of future possible selves. Research highlights that these specific psychological constructs are fundamental to prospective teachers’ professional development, adaptability, and subsequent instructional success (Bandura, 1997; Hamman et al., 2010; Tschannen-Moran and Woolfolk Hoy, 2001).

Despite the established importance of self-efficacy and possible selves, there is a significant gap in understanding how these constructs interact during the formative years of teacher education. Specifically, while it is known that self-efficacy influences career trajectories (Chesnut and Cullen, 2014; Rots et al., 2012), the exact mechanisms through which it shapes pre-service teachers’ expected and feared possible selves remain underexplored. Furthermore, the potential mediating role of teacher identity in this dynamic—how internalizing a professional role translates efficacy beliefs into future aspirations or fears—has not been sufficiently addressed in the literature. Without this knowledge, teacher education programs may lack targeted strategies to prevent early career “reality shock” or mitigate the negative impacts of feared possible selves when expectations differ from actual practice (Woolfolk Hoy and Spero, 2005).

To address this critical gap, the present study investigates the role of teacher identity as a mediator in the relationship between self-efficacy and both expected and feared possible selves among pre-service teachers. By examining these associations, this research aims to elucidate how developing a strong professional identity can bridge the gap between a teacher’s current confidence (self-efficacy) and their future-oriented cognitions (possible selves). Ultimately, the findings will provide actionable insights for educators and policymakers to design interventions that strengthen positive future selves and mitigate career-related fears in teacher preparation programs (Brown and Lent, 2017; Siwatu and Chesnut, 2014).

## Literature review

### Self-efficacy

The concept of self-efficacy refers to an individual’s confidence in their own ability to orchestrate and perform the necessary sequences of action to accomplish specific objectives (Bandura, 1997). People with high levels of this trait are assured in their personal competence and their grasp of the strategies required for task completion.

Though straightforward in its definition, teacher self-efficacy carries profound implications for the classroom. It refers to an educator’s belief in their ability to foster student learning and engagement, including when facing the challenge of teaching difficult or unmotivated students (Bandura, 1977; Maddux, 1995). Teachers’ sense of efficacy affects student outcomes, including achievement, motivation, and classroom behaviors (Bandura, 1997; Zee and Koomen, 2016). Within an educational setting, teacher self-efficacy refers to an educator’s personal conviction in their ability to successfully execute specific instructional tasks with a defined standard of quality, depending on the situation (Dellinger et al., 2008, p. 752).

Researchers examining preservice teachers found that increased self-efficacy indicates readiness to teach, highlighting the role self-efficacy may play in retaining teachers (Skaalvik and Skaalvik, 2010). Moreover, high level of self-efficacy supports active involvement in tasks in educational contexts (Huang et al., 2019). Significant issues in educational contexts impacting teacher self-efficacy have been identified in several key areas, including the ability to manage classroom environments effectively to facilitate and to engage students in the learning process (Siwatu, 2008, 2011; Tschannen-Moran and Woolfolk Hoy, 2001).

### Teacher identity

Teacher identity is fundamentally defined as a teacher’s inner perception of themselves as a professional, encompassing their core beliefs, values, and commitments regarding their educational role. Throughout this manuscript, the terms teacher identity, professional identity, and teacher professional identity are used interchangeably to denote this overarching construct. Rather than being a fixed entity, scholars conceptualize this identity as a multifaceted and evolving construct that is continuously shaped by the personal stories individuals tell about themselves (Beijaard et al., 2004; Rodgers and Scott, 2008). Complementing this internal view is the understanding that identity is also socially constructed, with its functions and expressions becoming meaningful through interaction with others (Goffman, 1959; Mead, 1934). Consequently, an individual’s identity emerges from a synthesis of the personal meanings they create and the meanings that are attributed to them within their social world (Beijaard, 1995). It is often shaped by one’s early childhood experiences, teacher role models, and the influence of family and significant others (Knowles, 1992; Sugrue, 1997). Additionally, professional identity can be expressed as a professional essence based on characteristics, experiences, values and beliefs (Kavrayıcı, 2020; Slay and Smith, 2011, p. 85). A person’s professional identity is one of the roles that differentiates and defines them. Defining one’s identity as “professional” is directly proportional to defining oneself as belonging to a profession or a professional group and the norms required by the profession. Therefore, teacher’s professional identity is founded upon the interplay between their social and individual identities, requiring interpretation through both individual and collective lenses (Mockler, 2011).

Teacher identity, which can be considered as a role performance, has also been addressed in the literature on both sociological and psychological grounds. From a sociological identity perspective, teacher identity or professional identity is the categorization, evaluation and comparison of the teaching profession within a particular professional group or community (Straub, 2002). A sociological examination of professional identity formation brings critical contextual factors to the forefront, including policies, institutional culture, school structures, and collective interactions with colleagues, students, and administrators (Cheng, 2008; Nguyen, 2008). Sociological factors can be classified as relational and contextual factors. Contextual factors consist of sociological factors that surround teachers and are established by the social structure. These factors include culture, institutions, schools, reform movements and policies (Flores and Day, 2006). Relational factors consist of the relationships that teachers establish with other individuals. These relationships include division of labor, collegiality, collegial cooperation, cohesion, and relationships with students (Hallman, 2007; McCormack et al., 2006).

Furthermore, psychological factors are also crucial in shaping professional identity. One of the key factors is motivation, encompassing both intrinsic and extrinsic drives. Intrinsic motivation, characterized by a teacher’s personal fulfillment from their work, is a key factor associated with the development of a stronger professional identity. Extrinsic factors, such as recognition or rewards, can further reinforce this identity (Deci and Ryan, 1985; Korthagen, 2004). Job satisfaction also plays a critical role, as teachers who are content with their work conditions and relationships tend to have a more positive view of their professional roles, which strengthens their identity and commitment to the profession (Day et al., 2007). Closely related is professional commitment, which reflects a teacher’s dedication to continuous development and engagement in the profession. A strong commitment to the profession often fosters a self-perception as a lifelong learner, an outlook that subsequently strengthens a teacher’s professional identity (Coladarci, 1992). Furthermore, emotional regulation and resilience contribute to professional identity by equipping teachers with strategies to navigate the emotional demands of their profession, such as managing disruptive student behaviors, coping with workload stress, or dealing with unmotivated students. Teachers who effectively regulate their emotions and exhibit resilience are better equipped to maintain a positive professional identity in the face of adversity (Day and Gu, 2014).

### Possible selves

Possible selves are explicitly defined as the future-oriented components of an individual’s self-concept, representing the specific versions of the self that one hopes to become, expects to become, or fears becoming (Markus and Nurius, 1986). Building on this definition, possible selves offer a way for individuals to articulate their hopes, dreams, and expectations, as well as the outcomes they want to avoid (Hardgrove et al., 2015; Kavrayıcı, 2022). Possible selves assist individuals in navigating transitions and changes, offering a way to alleviate the sense of limitation in their current choices (Plimmer and Schmidt, 2007). Introduced by Markus and Nurius (1986), possible selves theory has been a cornerstone for comprehending future-oriented cognition since the 1980s. It elucidates the process by which individuals envision their potential futures. Fundamentally, these possible selves act as “incentives for future behavior,” deriving from an individual’s interpretations of their past and present (Markus and Nurius, 1986, p. 955). People may imagine possible selves they aspire to become or fear becoming. By generating possible selves, individuals can predict who they are likely to be in the future (Oyserman and Lee, 2008).

Consequently, possible selves fulfill two critical roles: they provide a cognitive bridge from an individual’s current reality to their anticipated future (Oyserman et al., 2004), and they operate as a source of motivation, guiding behavior toward the realization of desired outcomes and the avoidance of feared ones. Moreover, possible selves may either build strong connection with their present self, highlighting continuity, or signify a complete departure from it. The amount and variety of possible selves vary depending on individual. Possible selves reflect both aspirations and future concerns. Hoped-for selves might encompass happiness, job satisfaction, and a healthy family. On the other hand, feared selves might involve loneliness, illness, and poverty (Dunkel and Anthis, 2001). Specifically in the context of teaching, expected possible selves might include becoming a highly professional and continuously learning educator, whereas a feared possible self could manifest as becoming an uncaring teacher, experiencing a loss of classroom control, or delivering uninspired instruction.

The theory of possible selves, originally developed by Markus and Nurius (1986), has evolved into a well-established and frequently applied construct in numerous areas of study. Its influence is especially prominent in both general education and teacher education research (Fletcher, 2000; Hong and Greene, 2011; Oyserman et al., 2006; Pizzolato, 2006).

### Conceptual framework and hypotheses development

#### Teacher identity and possible selves

The correlation between possible selves and teacher identity is evident in the significant role that identity exploration plays in generating possible selves, the influence of previous experiences on the development of future teacher identity and the evolving character of possible selves. In the context of teacher education, the possible selves of teacher candidates are significantly influenced by their pre-service teacher identity. This relationship between pre-service teacher identity and possible selves has been explored in various studies, highlighting the impact of past experiences and identity formation on future aspirations and fears (Hamman et al., 2010; Hong and Greene, 2011). The development of a pre-service teacher’s professional identity is an integrative process, drawing upon their personal history, academic training, and early practical experiences in the classroom. As noted by Beijaard et al. (2004), the identity of a teacher candidate is a multifaceted construct built upon personal stories, and these narratives are critical in forming the individual’s possible selves.

Dunkel and Anthis (2001) and Dunkel (2000) emphasize that the past selves of teacher candidates significantly influence their envisioned possible selves. The formative experiences during teacher training, including interactions with mentors, peers, and students, support the formation of a teacher’s professional identity. These experiences create a foundation upon which future aspirations and concerns are built. Hamman et al. (2010) describe how possible selves are dynamic, continuously reshaped by new information and experiences. This process of imagining and re-imagining future selves is affected by the evolving identity of pre-service teachers. As teacher candidates gain more practical experience, their comprehension of the current and future teacher identity becomes clearer.

They also emphasized that the theory of possible selves illustrates how future-oriented thinking is driven by self-relevant goals and contributes to understanding one’s sense of identity. More explicitly, connecting present self to the possible self can enhance welfare and promote self-development, thereby guiding future behavior. When individuals feel their possible selves are aligned with their current selves, they can maintain a robust sense of identity (Nurra and Oyserman, 2018). Longitudinal studies provide valuable insights into the association between identity investigation and the generation of possible-selves. A study by Dunkel and Anthis (2001) demonstrated that the extent to which a person explores their identity directly influences the range and number of possible selves they can conceive. This suggests that the ongoing process of identity formation during teacher training directly impacts the formation of future-oriented possible selves.

Moreover, teacher training curriculum has a central position in shaping the professional identity of teacher candidates. Research by Brown and Lent (2017) and Siwatu and Chesnut (2014) highlights that fears, hopes, and aspirations of pre-service teachers are crucial in understanding their professional identity development. By addressing these aspects, teacher preparation programs can enhance the construction of a coherent and affirmative professional identity, which in turn influences the possible selves of pre-service teachers. In essence, the theory of possible selves provides a valuable lens for examining the reciprocal relationship between future-oriented cognition and the ongoing development of identity. Oyserman et al. (2006) and Oyserman et al. (2004) studied the association between possible selves and academic performance suggesting that envisioned possible selves can predict future behaviors and achievements. To understand this dynamic fully, it is essential to clearly distinguish between expected and feared possible selves within the teaching context. Expected possible selves represent the positive future states a pre-service teacher aspires to achieve, such as becoming a highly competent, inspiring, and continuously developing educator. In contrast, feared possible selves represent the negative, undesired future outcomes they dread, such as becoming an uncaring teacher, delivering uninspired instruction, or losing control of the classroom (Hamman et al., 2013).

A strong professional identity provides pre-service teachers with a clear cognitive roadmap and a stable self-concept, which facilitates the construction of positive, expected possible selves. Furthermore, a well-established teacher identity acts as a psychological buffer against professional anxieties, reducing the salience of negative, feared possible selves. Consequently, it is logically expected that as pre-service teachers develop a more robust professional identity, their positive future aspirations are reinforced, while their future-oriented fears are mitigated. Based on this theoretical framework, the following hypotheses are proposed:

*H*1. Teacher identity is positively related to expected possible selves.

*H*2. Teacher identity is negatively related to feared possible selves.

#### Self-efficacy and possible selves

The professional growth of pre-service teachers is deeply informed by two key psychological constructs: self-efficacy and possible selves (Narayanan and Ordynans, 2022; Siwatu and Chesnut, 2014). Rooted in Bandura (2001) social cognitive theory, self-efficacy refers to an individual’s conviction in their ability to successfully perform specific actions, a belief shaped by experiential, social, and environmental influences. Complementing this, the concept of possible selves (Markus and Nurius, 1986) captures the spectrum of an individual’s imagined futures, which are populated by aspirations and apprehensions about who they might become. For decades, educational researchers have pursued two parallel lines of inquiry: the statistical measurement of teacher turnover and a qualitative exploration of the motivations that drive retention and attrition. It has been suggested that teachers’ decisions regarding entering, staying in, or leaving the profession stem from both the immediate and mediated effects of work-related beliefs, including self-efficacy and outcome expectations as well as their interests and short-range or long-range career targets (Siwatu and Chesnut, 2014). Moreover, the correlation between possible selves and self-efficacy has been emphasized in the literature, further supporting the notion that teachers’ perceptions about their competencies and future outcomes significantly shape their career trajectories. Furthermore, the transition from pre-service to in-service teaching is often accompanied by a ‘reality shock’ when initial expectations conflict with the actual demands of the classroom. In this critical phase, robust self-efficacy beliefs and well-defined expected possible selves serve as vital psychological resources, helping novice teachers navigate these discrepancies and reducing the likelihood of early career attrition (Voss and Kunter, 2020; Woolfolk Hoy and Spero, 2005).

A substantial body of research indicates that a teacher’s self-efficacy beliefs are a strong predictor of their professional performance, actions, and overall conduct in the classroom (Bandura, 1997; Klassen and Chiu, 2011; Zee and Koomen, 2016). In general, self-efficacy is shaped by mastery experiences, vicarious experiences (Bandura, 1977). A teacher’s efficacy beliefs are significantly bolstered by mastery experiences, which are defined as the perceived successful performance of a teaching duty. This enhancement in self-assurance promotes a positive outlook on the outcome of future teaching efforts (Tschannen-Moran et al., 1998). Vicarious experiences, like observing the successful teaching performances of others, also contribute to developing self-efficacy, as pre-service teachers are able to shape their behaviors based on those they have observed. These observations not only influence current teaching practices but also help shape teachers’ possible selves—their envisioned future abilities and roles—by providing a framework for what they aspire to achieve in their professional development. Through these vicarious experiences, teachers begin to imagine themselves adopting similar successful strategies, which strengthens their belief in their future capacity to handle challenges and grow in their profession (Schunk and Zimmerman, 2012).

These origins of self-efficacy play a crucial role within the teaching context because they not only shape individual confidence but also influence key professional outcomes. It has become one of the most frequently studied factors thought to impact both preservice and in-service teachers’ commitment, burnout, and their readiness to embrace and implement educational reforms (Tschannen-Moran and Woolfolk Hoy, 2007; Chesnut and Cullen, 2014) Educators who possess high levels of self-efficacy demonstrate a stronger dedication to teaching, a greater capacity to withstand professional exhaustion, and an increased openness to implementing new practices. These attributes establish self-efficacy as an indispensable component for successful teacher development and the advancement of education reform.

Several other studies provide empirical evidence for the correlation between self-efficacy and possible selves in pre-service teachers. Hamman et al. (2010) found that teachers’ consideration of their future selves serves as both a motivator for action and a benchmark for self-evaluation. The ability to envision positive future selves can drive pre-service teachers to strive for improvement and engage more deeply in their professional development. Similarly, Woolfolk Hoy and Spero (2005) found that the self-efficacy of pre-service teachers fluctuated when faced with the realities of teaching, suggesting that those with higher self-efficacy developed more positive perspectives on their future teaching roles. Schunk and Zimmerman (2012) emphasized the effect of self-efficacy on the goals of individuals set, noting that high levels of self-efficacy among teacher candidates are associated with the formation of more constructive possible selves. Additionally, Zee and Koomen (2016) examined the association between self-efficacy and student engagement via longitudinal study, revealing that high level of self-efficacy teachers enhance student participation, thereby shaping their professional identities. Lastly, Efthymiadou et al. (2025) explored the connections among self-efficacy, emotional intelligence, and possible selves, finding that elevated self-efficacy levels correlate with more positive possible selves. Collectively, these findings underscore the importance of teacher training programs actively fostering the self-efficacy beliefs of teacher candidates by providing structured opportunities for mastery and vicarious experiences. Specifically, through hands-on practicum placements where candidates can successfully execute teaching tasks (mastery), and by observing experienced mentor teachers (vicarious learning), these programs can effectively build candidates’ confidence. This intentional development of self-efficacy can lead to the formulation of more positive and constructive possible selves, ultimately contributing to their professional growth and effectiveness. Drawing on the empirical literature that establishes a link between self-efficacy and possible selves, the following hypothesis is proposed:

*H*3. There is a positive relationship between self-efficacy and expected possible selves.

*H*4. There is a negative relationship between self-efficacy and feared possible selves.

### Self-efficacy and teacher identity

The association between self-efficacy and teacher identity—consistently defined as a teacher’s inner perception of their professional self—is firmly supported by the literature. Empirical research highlights the crucial role of self-efficacy in the development of this internal professional perception and dedication to teaching (Beauchamp and Thomas, 2009; Canrinus et al., 2012). Bandura's (1997) seminal work, supported by later studies, positions self-efficacy as a key factor that enhances a teacher’s feeling of professional capability while simultaneously strengthening their dedication to teaching (Bogler and Somech, 2004). Empirical evidence links self-efficacy to core professional outcomes; for instance, Canrinus et al. (2012) found it to be a significant enhancer of professional identity and commitment. Similarly, Beauchamp and Thomas (2009) argue for the inseparability of identity and self-efficacy, emphasizing the latter’s role in shaping teacher autonomy, decision-making, and growth. As teacher candidates and teachers develop stronger self-efficacy, they become more confident in adopting innovative practices, overcoming obstacles, and fostering student success. Crucially, this heightened confidence in their capabilities allows them to internalize a positive professional self-image, linking these external behaviors directly to their inner perception of self (Beijaard et al., 2000). Consequently, teacher self-efficacy is deeply interwoven with the development of a professional identity, influencing both immediate teaching practices and long-term career trajectories.

The interplay between self-efficacy and teacher identity is also evident in how teachers respond to challenges and engage in professional learning. A robust professional identity—defined here as a stable, confident, and well-integrated internal perception of oneself as an effective educator—reinforced by strong self-efficacy, empowers teachers to become agents of change; they demonstrate a greater propensity for innovation, a stronger commitment to implementing reforms, and enhanced resilience when confronting professional challenges (Beijaard et al., 2000; Beauchamp and Thomas, 2009). Self-efficacy and professional identity forms a critical foundation for the development of pre-service teachers. A well-established body of research indicates that those who enter the profession with strong efficacy beliefs are more inclined to adopt a self-perception as competent educators, a crucial step that subsequently consolidates a robust and enduring professional identity (Beauchamp and Thomas, 2009; Canrinus et al., 2012). This relationship is bidirectional, with a well-established professional identity enhancing self-efficacy, creating a positive cycle of development. Pre-service teacher identity, as described by Day et al. (2006), is influenced by self-efficacy and shaped by personal beliefs of pre-service teachers, external factors, their social interactions, and the teaching environment. A pre-service teacher’s confidence in core competencies—such as classroom management, student engagement, and instructional delivery—provides a foundation for envisioning a successful future self, a process that directly reinforces the development of their professional identity (Beijaard et al., 2004). The findings of Rots et al. (2012) and Chesnut and Cullen (2014) position self-efficacy as a critical determinant of a teacher’s career path, influencing both their commitment to join and their resolve to remain in the field, thereby highlighting its significance in professional identity formation.

Longitudinal research indicates that while self-efficacy can fluctuate during teacher preparation, sustaining higher levels is linked to a stronger professional identity (Tschannen-Moran and Woolfolk Hoy, 2007). Furthermore, a perceived capability to overcome challenges fosters a deeper connection to the teaching role and facilitates the construction of clearer, more optimistic future professional visions (Cardelle-Elawar et al., 2007). Synthesizing this evidence, the following hypothesis is proposed:

*H*5. There is a positive relationship between self-efficacy and teacher identity.

### Linking self-efficacy and possible selves through teacher identity as a mediating variable

Empirical evidence consistently underscores a significant positive relationship between teacher identity and self-efficacy (Beauchamp and Thomas, 2009; Canrinus et al., 2012), as well as between self-efficacy and the formation of expected possible selves (Hamman et al., 2010; Woolfolk Hoy and Spero, 2005). Teacher identity can be understood as both an individual and socially constructed phenomenon that evolves through personal experiences, social interactions, and professional contexts (Beijaard et al., 2004). Grounded in social cognitive theory (Bandura, 1997), self-efficacy is a primary driver of human motivation and behavior. For educators, the development of a robust sense of efficacy is instrumental in consolidating a strong professional identity. This fortified identity, in turn, shapes their future-oriented cognitions, enabling the construction of optimistic and professionally ambitious possible selves (Nurra and Oyserman, 2018). In this regard, previous studies have demonstrated that teacher identity plays a significant mediating role in the relationship between self-efficacy and various professional outcomes, such as burnout (Lin et al., 2022), achievement and school persistence (Oyserman et al., 2006), and beliefs about teaching (Assante and Cuza, 2020).

According to Hamman et al. (2010), a pre-service teacher’s sense of efficacy directly influences their professional expectations, and the self-perception of being a competent practitioner is a key factor in cultivating a stronger professional identity. By demonstrating that self-efficacy affects both instructional practice and enduring professional identity, Tschannen-Moran and Woolfolk Hoy (2007) provide a foundation for hypothesizing that teacher identity mediates the link between self-efficacy and possible selves. Furthermore, research has established connections between self-efficacy and academic outcomes (Zee and Koomen, 2016), emotional regulation (Efthymiadou et al., 2025), and identity development (Beijaard et al., 2004), all of which are influenced by teachers’ evolving sense of identity.

To date, however, there remains limited literature directly exploring the mediating role of teacher identity in the relationship between self-efficacy and possible selves, particularly in pre-service teacher contexts. This lack of knowledge is highly problematic; without understanding how professional identity translates efficacy beliefs into future expectations, teacher education programs remain ill-equipped to design targeted interventions that prevent early-career attrition and “reality shock.” Bridging this gap is not just an academic exercise, but a practical necessity. Understanding this mediating mechanism provides actionable insights for educators and policymakers to develop comprehensive models of teacher growth.

### Theoretical rationale for the mediation model

While empirical evidence highlights the interrelations among self-efficacy, teacher identity, and possible selves, establishing a tightly integrated theoretical rationale for their specific directional sequence is essential. Rather than viewing these constructs as merely parallel, this study posits a sequential mediation model grounded in the developmental trajectory of pre-service teachers.

First, regarding the antecedent role of self-efficacy on teacher identity: Although the relationship between self-efficacy and professional identity may become reciprocal as teachers gain years of experience, during the formative pre-service years, self-efficacy functions as a foundational antecedent. Grounded in Bandura (1997) social cognitive theory, early mastery and vicarious experiences during teacher education initially build a sense of teaching capability. This fundamental belief in one’s competence is a necessary psychological prerequisite for the deeper, more complex internalization of the professional role (Beauchamp and Thomas, 2009). In essence, before a pre-service teacher can confidently claim the identity of “I am a teacher,” they must first establish the belief that “I can teach.” Therefore, self-efficacy acts as the primary catalyst that shapes and solidifies teacher identity.

Second, regarding the influence of teacher identity on possible selves: According to the foundational theory by Markus and Nurius (1986), possible selves are direct future projections of the current self-concept. Therefore, a well-defined current teacher identity provides the essential cognitive schema from which pre-service teachers can envision their futures. A strong professional identity acts as an anchor in the present, enabling candidates to articulate realistic, specific, and attainable future aspirations, while also providing a framework to recognize and confront potential professional threats (Hamman et al., 2010). Thus, identity serves as the bridge connecting current capabilities to future-oriented cognitions.

Third, regarding the differential strength and nature of mediation for expected versus feared possible selves: The mechanisms through which teacher identity mediates these two dimensions are theoretically distinct. Expected possible selves are closely and directly linked to one’s perceived capability (self-efficacy); if one feels capable, one naturally expects to succeed. Thus, self-efficacy exerts both direct and indirect effects (suggesting partial mediation) on positive future visions. Conversely, feared possible selves—such as the dread of losing classroom control or becoming an uncaring educator—represent deep-seated, existential professional anxieties. High self-efficacy alone may not suffice to completely eliminate these pervasive fears. Instead, efficacy must be translated into a secure, internalized professional identity, which then serves as a crucial psychological buffer against negative future projections (Oyserman and Fryberg, 2006). Consequently, teacher identity is theoretically expected to exert a stronger, potentially full mediating effect in the relationship between self-efficacy and feared possible selves.

By integrating these insights, this model ensures that novices are guided by expected possible selves rather than paralyzed by feared ones. Following this theoretical rationale, the following hypotheses were presented:

*H*6. Teacher identity mediates the relationship between self-efficacy and expected possible selves.

*H*7. Teacher identity mediates the relationship between self-efficacy and feared possible selves.

## Method

### Research design

The present study is situated within a quantitative paradigm and adopts a cross-sectional research design. In a cross-sectional approach, data is gathered from a specific population at a single point in time (Fraenkel et al., 2012, p. 394).

### Participants and procedures

This study included 511 pre-service teachers from a university in the Central Anatolia region of Türkiye. The sample consisted of 360 females (70.5%) and 145 males (28.4%), while 6 (1.2%) participants did not specify their gender. Participants’ ages ranged from 18 to 45 (M = 21.04, SD = 2.07). In the Turkish educational system, teacher education is a standard four-year undergraduate program. Accordingly, in terms of academic year, 66 (12.9%) were freshmen (first year), 175 (34.2%) sophomores (second year), 159 (31.1%) juniors (third year), and 102 (20%) seniors (fourth year); 9 (1.8%) did not report their year level. Participants were recruited using a convenience sampling method rather than strict random sampling. Although the survey was hosted online via Google Forms, data collection was conducted in person during regular educational course hours. The survey link was distributed by the primary researcher, who personally supervised all data collection sessions across the various courses to ensure consistency. Students accessed the questionnaire via their personal devices (smartphones or laptops). Written informed consent was obtained from all participating pre-service teachers prior to their involvement in the study. The consent procedure involved providing a detailed information sheet explaining the study’s purpose, procedures, potential risks and benefits, confidentiality assurances, and the voluntary nature of participation. Participants were given approximately 15 to 20 min to read the information, ask questions to the attending researcher, and provide their consent before completing the survey. The survey instrument comprised the following self-report measures: the Teacher’s Sense of Efficacy Scale, the Teacher Identity Scale, the New Teacher Possible Selves Questionnaire, and a Demographic Characteristics Survey. The demographic profile of the participants is presented in Table 1.

**Table 1:** Participant demographics.

Variables	Categories	f	%
Gender	Female	360	70.5
	Male	145	28.4
	Missing	6	1.2
Seniority	Freshman	66	12.9
	Sophomore	175	34.2
	Junior	159	31.1
	Senior	102	20
	Missing	9	1.8
Teaching Experience	Yes	162	31.7
	No	342	66.9
	Missing	7	1.4
Department	Primary School Education	46	9
	Primary School Mathematics Teaching	137	26.8
	Special Education	101	19.8
	Foreign Language Teaching	57	11.2
	Arts and Crafts Education	13	2.5
	Pre-School Education	123	24.1
	Social Science Education	28	5.5
	Missing	6	1.2
Total		511	100

### Measures

The data collection instruments in this study were administered in their original, validated formats to preserve their established psychometric properties. Consequently, the measures employ different Likert-scale response points (e.g., 9-point, 5-point, and 6-point), which, while not optimal for uniform scaling, ensures the validity and reliability of the specific adaptations used.

#### Teacher’s sense of efficacy scale

The Teacher’s Sense of Efficacy Scale, originally developed by Tschannen-Moran and Woolfolk Hoy (2001), was used in its Turkish adaptation by Çapa et al. (2005). This 24-item instrument employs a 9-point Likert scale, ranging from 1 (not at all) to 9 (a great deal), to measure teachers’ perceived efficacy. The scale comprises three 8-item subscales: Efficacy in Student Engagement, Efficacy in Instructional Strategies, and Efficacy in Classroom Management. A sample item from the scale is, “How much can you do to motivate students who show low interest in schoolwork?.” In the Turkish adaptation, the internal consistency was high (*α* = 0.95). In the present study, the scale demonstrated high reliability, with a Cronbach’s alpha of α = 0.93.

#### Teacher identity scale

Teacher identity was measured using the Teacher Identity Scale, originally developed by Friesen and Besley (2013) and subsequently adapted into Turkish by Arpacı and Bardakçı (2015). The instrument consists of 17 items rated on a 5-point Likert scale, ranging from 1 (strongly disagree) to 5 (strongly agree). Total scores range from 17 to 85, with higher scores indicating a stronger sense of professional identity among pre-service teachers. This measure operationalizes “identity” as the pre-service teachers’ internal perception and self-categorization as professionals, capturing their cognitive and emotional attachment to the teaching role. A sample item from the scale is, “I feel comfortable identifying myself as a teacher.” The scale demonstrated high reliability in its original development (α = 0.87) and the Turkish adaptation (α = 0.91). In the present study, the internal consistency was also high, with a Cronbach’s alpha of α = 0.89.

#### New teacher possible selves questionnaire

The New Teacher Possible Selves Questionnaire, developed by Hamman et al. (2013) and adapted into Turkish by Tatli-Dalioglu and Adiguzel (2015), was used to assess future-oriented self-perceptions. The instrument comprises two distinct scales: the Expected Teacher Possible Selves Scale (9 items, 2 factors: Professionalism and Learning to Teach) and the Feared Teacher Possible Selves Scale (9 items, 3 factors: Uninspired Instruction, Loss of Control, and Uncaring Teacher). Responses are recorded on a 6-point Likert scale, from 1 (definitely not expecting/fearing) to 6 (definitely expecting/fearing). A sample item for the Expected scale is, “I expect to be a teacher who helps students develop positive attitudes” and for the Feared scale, “I fear becoming a teacher who loses control of the classroom.” In the Turkish adaptation, the internal consistency was high for both the Expected (α = 0.87) and Feared (α = 0.92) scales. In the present study, the Cronbach’s alpha coefficients were α = 0.91 for the Expected scale and α = 0.93.

### Data analysis

Descriptive statistics and bivariate correlations between the variables were presented before testing the hypothesized models (see Table 2). Then, two separate simple mediation models were specified to test the hypothesized effects. Specifically, the first model tested the mediating role of teacher identity (mediator) in the relationship between self-efficacy (predictor) and expected possible selves (outcome). The second model tested the mediating role of teacher identity (mediator) in the relationship between self-efficacy (predictor) and feared possible selves (outcome). The hypothesized mediation models were tested using SPSS version 22.0 and the PROCESS macro (version 3.3) developed by Hayes (2018). Given that the PROCESS macro utilizes ordinary least squares (OLS) regression-based path analysis for manifest variables, traditional global model fit indices (e.g., CFI, RMSEA) are not computed. Instead, the adequacy of the models is evaluated based on the statistical significance of the individual pathways. The analysis estimated the total, direct, and indirect effects in accordance with recent methodological guidelines for causal mediation analysis (Li et al., 2023; Nguyen et al., 2021), generating 95% bias-corrected bootstrap confidence intervals based on 5,000 bootstrap resamples. All path coefficients are reported as standardized beta (*β*) weights. Prior to the main analyses, the assumption of univariate normality was assessed by examining skewness and kurtosis statistics for all study variables (see Table 2). The univariate normality of all variables was confirmed, with skewness and kurtosis values falling within the acceptable range of ±2 (George and Mallery, 2013). Furthermore, the assumptions of multivariate normality, linearity, and the absence of multicollinearity were also examined and met prior to conducting the mediation analysis. No outliers were detected in the dataset. Since VIF values of the data were lower than 5 and tolerance values were greater than 0.10 there was not multicollinearity (Cohen et al., 2003). Furthermore, Durbin-Watson test statistics fell within the acceptable range of 1 to 3, indicating no autocorrelation was present in the data (Field, 2009).

**Table 2:** Relationships among the variables.

	M	SD	Skewness	Kurtosis	1	2	3	4
1. Self-efficacy	6.51	1.05	−0.171	−0.004	-			
2. Teacher identity	3.83	0.62	−0.705	0.792	0.548^*^	-		
3. Expected possible selves	5.04	0.63	−0.640	0.788	0.493^*^	0.531^*^	-	
4. Feared possible selves	3.44	1.28	0.050	−0.973	−0.161^*^	−0.192^*^	−0.077	-

*Correlation is significant at the 0.01 level.

## Results

In all analyses, the variables (self-efficacy, teacher identity, expected possible selves, and feared possible selves) were modeled as manifest indicators by calculating the composite mean scores of their respective scale items. Findings from the Pearson correlation analysis revealed statistically significant positive associations among self-efficacy and teacher identity (*r* = 0.548, *p* < 0.01) and expected possible selves (*r* = 0.493, *p* < 0.01) while it had significantly negative correlations with feared possible selves (*r* = −0.161, *p* < 0.01). Teacher identity had significantly positive correlations with expected possible selves (*r* = 0.531, *p* < 0.01), while it had significantly negative correlations with feared possible selves (*r* = −0.192, *p* < 0.01). Moreover, expected possible selves had significantly negative correlations with (*r* = −0.077, *p* < 0.01) feared possible selves (Table 2). All bivariate correlation coefficients were below 0.70, indicating the absence of severe multicollinearity among the study variables.

**Figure 1:**
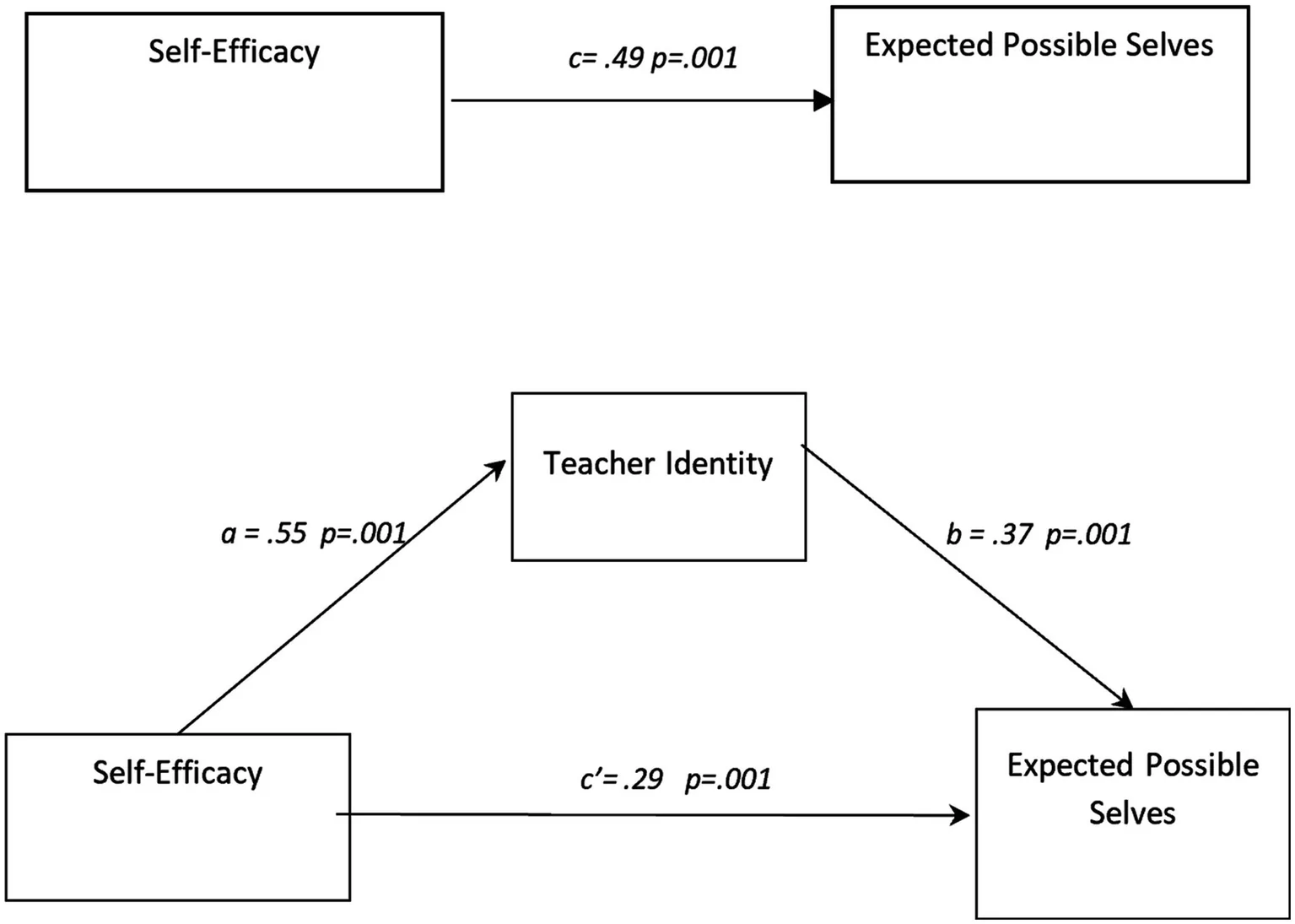
Simple mediation model testing the mediating effect of teacher identity.

The conceptual diagram of the basic mediation model is presented in Figure 1. The analyses conducted for the first hypothesized model presented in Figure 1 demonstrated that the total effect of self-efficacy on expected possible selves was statistically significant [*β* = 0.49, t(509) = 12.79, *p* < 0.001], suggesting that pre-service teachers with higher self-efficacy scores are more likely to report higher levels of expected possible selves. Secondly, path (a) from self-efficacy to teacher identity was positive and statistically significant [*β* = 0.55, t(509) = 14.79, *p* < 0.001], indicating that pre-service teachers with higher self-efficacy scores tend to report stronger teacher identities. Third, the coefficient between teacher identity and expected possible selves in path (b) was statistically significant [*β* = 0.37, t(509) = 8.64, *p* < 0.001], indicating that pre-service teachers with stronger teacher identities are more likely to report higher levels of expected possible selves. Finally, as shown in Figure 1, the path coefficient between self-efficacy and expected possible selves [*β* = 0.29, t(508) = 6.71, *p* < 0.001] remained statistically significant, although it was reduced when teacher identity was included in the model as a mediating variable.

The bootstrapping results indicated that the indirect effect of self-efficacy on expected possible selves through teacher identity was statistically significant (*β* = 0.12, 95% CI [0.08, 0.16]). As the confidence interval did not include zero, it can be concluded that the findings are consistent with a mediation model supporting an indirect association pattern (Preacher and Hayes, 2004). Furthermore, this indirect association is partial, as the direct effect of self-efficacy on expected possible selves remained significant even with the mediator included in the model.

**Figure 2:**
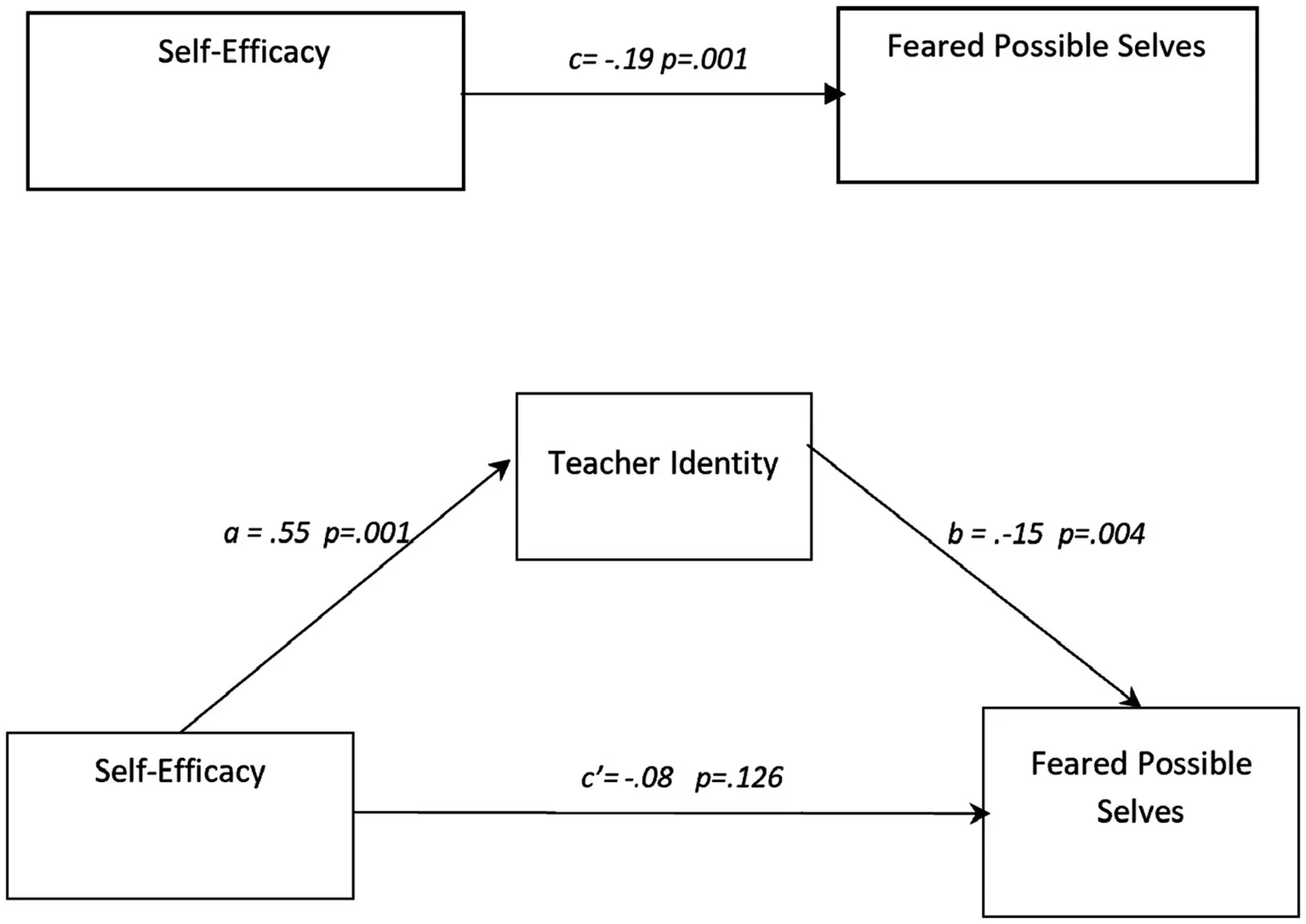
Second simple mediation model testing the mediating effect of teacher identity.

For the second hypothesized model testing H7, the analyses demonstrated that the total effect of self-efficacy on feared possible selves was statistically significant [*β* = −0.19, t(509) = −3.67, *p* < 0.001], indicating that pre-service teachers with higher self-efficacy scores tend to report lower levels of feared possible selves. The analysis for path (a) ordinarily produced the same statistically significant results as in first hypothesized model [*β* = 0.55; t (509) = 14.79, *p* = 0.001]. Moreover, the coefficient between teacher identity and feared possible selves in path (b) was statistically significant [*β* = −0.15, t(509) = −2.85, *p* = 0.0014], indicating that pre-service teachers with higher levels of teacher identity tend to report lower levels of feared possible selves. Finally, as presented in Figure 2, the path coefficient between self-efficacy and feared possible selves [*β* = −0.08, t(508) = −1.53, *p* = 0.126] was not statistically significant when teacher identity was included as a mediating variable in the model.

According to the bootstrapping results, the mediation analysis indicated that the indirect pathway from self-efficacy to expected possible selves through teacher identity was statistically significant (bootstrap estimate = −0.09, 95% CI = −0.16, −0.03) and did not contain zero. This result indicated that self-efficacy had a statistically significant indirect association with feared possible selves through teacher identity. Given the cross-sectional nature of the data, this represents an associational indirect relationship rather than a causal temporal mechanism. These results reveal that the findings are consistent with a mediation model, supporting a full indirect association pattern between self-efficacy and feared possible selves through teacher identity, as the effect of the independent variable on the dependent variable became nonsignificant when the mediator was included.

## Discussion

This study aimed to examine the associational relationships between self-efficacy, expected and feared possible selves, and the mediating role of teacher identity. In exploring these dynamics, the first hypothesis testing the positive relationship between teacher identity and expected possible selves was supported. This finding corroborates the research of Hamman et al. (2013), which established that a well-defined professional identity provides a cognitive framework for teachers to construct attainable visions of their future successes. Furthermore, as Nurra and Oyserman (2018) emphasize, a strong professional identity offers clarity and direction, allowing pre-service teachers to develop more realistic and optimistic expectations. Consequently, when pre-service teachers hold a confident teacher identity, they tend to envision a rewarding professional future, which reinforces their current commitment to teaching (Beijaard et al., 2004).

In addition to fostering positive expectations, a robust teacher identity was found to be significantly and negatively associated with feared possible selves, supporting the second hypothesis. This finding is consistent with Dunkel and Anthis (2001), who argue that a well-established and stable identity helps mitigate fears about negative future outcomes. Pre-service teachers with a clear understanding of their professional trajectory possess higher self-assurance, making them less likely to focus on feared possible selves. Conversely, as Hong and Greene (2011) highlight, teachers with an underdeveloped sense of identity are more prone to fears about their professional future. Therefore, a well-defined identity not only supports expected possible selves but actively reduces the salience of negative career aspirations (Oyserman and Fryberg, 2006).

Self-efficacy also demonstrated significant direct associations with both dimensions of possible selves, supporting the third and fourth hypotheses. Specifically, there was a positive relationship between self-efficacy and expected possible selves, alongside a negative relationship with feared possible selves. These results align with Tatli-Dalioglu and Adiguzel (2016), who reported similar correlation patterns among teacher candidates. The literature consistently positions self-efficacy as a critical predictor of performance (Bandura, 1997; Klassen and Chiu, 2010), extending its influence to the formation of future goals (Brady, 2011). As individuals’ self-efficacy increases, their expected possible selves flourish, while the inhibitory effects of feared possible selves—which can diminish professional agency (Yuan, 2016)—tend to decrease.

Furthermore, the fifth hypothesis was supported, indicating a strong positive relationship between self-efficacy and teacher identity. This finding aligns with existing empirical research demonstrating that self-efficacy serves as a critical component in shaping teachers’ professional identities. Beauchamp and Thomas (2009) discuss how self-efficacy strengthens teachers’ sense of agency, contributing to a clearer and more stable teacher identity. Similarly, Canrinus et al. (2012) found that teachers with high self-efficacy report stronger professional identities and greater professional commitment. Thus, teachers equipped with high self-efficacy are better prepared to overcome classroom challenges, which reinforces their professional identity and encourages the development of positive future perspectives (Tschannen-Moran and Woolfolk Hoy, 2007).

The sixth and seventh hypotheses investigated the mediating role of teacher identity in the relationships between self-efficacy and possible selves. The findings supported both hypotheses, revealing that the data are consistent with a mediation model wherein teacher identity supports a partial indirect association pattern between self-efficacy and expected possible selves (H6), and a full indirect association pattern between self-efficacy and feared possible selves (H7). These results indicate that self-efficacy not only has a direct association with how pre-service teachers envision their futures but also exerts a significant indirect effect by strengthening their professional identity. This finding is consistent with foundational studies by Canrinus et al. (2012) and Beauchamp and Thomas (2009), and is further corroborated by recent empirical research (Sun et al., 2025; Zarrinabadi et al., 2023), which demonstrates that self-efficacy serves as a fundamental predictor in cultivating a robust and positive professional identity among pre-service and early-career teachers. Specifically, higher self-efficacy fosters a more secure teacher identity, which in turn enhances positive career expectations. This mechanism aligns with the perspectives of Hamman et al. (2010) and Nurra and Oyserman (2018), and is echoed in recent qualitative explorations by de Bruin (2025), who notes that teachers’ current identity perceptions directly shape their future goals and evolving possible selves. Furthermore, by fully mediating the relationship with feared possible selves, teacher identity acts as a crucial psychological buffer. As suggested by earlier works (Dunkel and Anthis, 2001; Oyserman and Fryberg, 2006) and reinforced by recent evidence showing professional identity as a vital protective factor against career-related stress and negative emotional outcomes (Lin et al., 2022; Zarrinabadi et al., 2023), a clear and confident identity significantly reduces the salience of negative career aspirations and mitigates fears of professional failure. Ultimately, these findings advance the current literature by demonstrating that developing a strong professional identity—fueled by self-efficacy—is an essential mechanism for cultivating positive future visions and mitigating professional anxieties in pre-service teachers (Tschannen-Moran and Woolfolk Hoy, 2007).

### Limitations

This study has several limitations that should be considered when interpreting the findings. First, a cross-sectional survey design was implemented. The primary consequence of this design is that it precludes the ability to establish temporal precedence or cause-and-effect relationships among self-efficacy, teacher identity, and possible selves. Therefore, the mediation results must be strictly interpreted as associational indirect effects rather than causal mechanisms (Cain et al., 2018). Furthermore, cross-sectional mediation models cannot control for baseline differences, which could potentially distort the estimated indirect effects (Schuler et al., 2025). Future research should utilize longitudinal or experimental designs to verify the temporal sequence of these constructs. Second, data were collected exclusively through self-report measures, which may introduce common method bias and social desirability effects. Incorporating multiple data sources, such as observational data or mentor evaluations, could strengthen future investigations. Third, the study explored a mediating factor but did not test potential moderating variables. Future studies should consider how contextual or demographic factors might moderate these relationships. Finally, the participants were recruited from a single university in Türkiye. Consequently, the findings may lack generalizability to pre-service teachers in different cultural, geographic, or educational contexts. Replicating this study across diverse institutional settings is recommended to enhance the external validity of the results.

## Conclusion

Despite its limitations, this study makes significant contributions to understanding the role of teacher identity. By examining the relationship between self-efficacy and possible selves among pre-service teachers in Türkiye, this research highlights how teacher identity mediates this relationship. Specifically, the findings are consistent with a mediation model demonstrating that teacher identity supports a partial indirect association pattern when related to expected possible selves, while it supports a full indirect association pattern in the relationship between self-efficacy and feared possible selves. Although the cross-sectional design precludes drawing definitive causal conclusions for practice, these findings highlight highly relevant areas for potential educational support. Tentatively, these results suggest that teacher education programs might explore specific strategies to enhance self-efficacy as a foundational step. For example, providing pre-service teachers with structured mastery experiences (such as micro-teaching or extended practicums), opportunities for observational learning through mentorship programs, and continuous constructive feedback could be highly beneficial. By supporting self-efficacy through these practical examples, educators can help cultivate a more robust professional identity, which in turn may foster expected possible selves and mitigate the intensity of feared possible selves.

This research not only enhances our understanding of the complex interplay between self-efficacy, teacher identity, and possible selves but also offers practical insights for teacher education programs. By focusing on building and developing teacher identity during these programs, educators can better support pre-service teachers in fostering both self-efficacy and positive future selves. Prioritizing self-efficacy as a foundational element in teacher identity formation—through mastery experiences, observational learning, and constructive feedback—can lead to the development of resilient and committed teachers. Ultimately, the findings underscore that supporting pre-service teachers’ self-efficacy and professional identity is a crucial mechanism for cultivating optimistic expected possible selves and mitigating professional fears during their teacher education.

## Data Availability

The original contributions presented in the study are included in the article/supplementary material, further inquiries can be directed to the corresponding author.

## Author’s note

Brief preliminary findings of this study were presented at the ISNITE Congress.
